# Single-cell transcriptomic analysis of eutopic endometrium and ectopic lesions of adenomyosis

**DOI:** 10.1186/s13578-021-00562-z

**Published:** 2021-03-08

**Authors:** Zhiyong Liu, Zhonghua Sun, Hongyun Liu, Weipin Niu, Xin Wang, Na Liang, Xin Wang, Yanfei Wang, Yaxin Shi, Li Xu, Wei Shi

**Affiliations:** 1 Central Laboratory, The Affiliated Hospital of Shandong University of Traditional Chinese Medicine, No. 16369 Jingshi Road, Jinan, 250014 Shandong China; 2 Medical Department, The Affiliated Hospital of Shandong University of Traditional Chinese Medicine, No. 16369 Jingshi Road, Jinan, 250014 Shandong China; 3 Department of Gynecology, Linyi Central Hospital, No. 17 Jiankang Road, Yishui, 276400 Shandong China; 4 Grade Three Laboratory of Traditional Chinese Medicine Preparation of National Administration of Traditional Chinese Medicine, The Affiliated Hospital of Shandong University of Traditional Chinese Medicine, No. 16369 Jingshi Road, Jinan, 250014 Shandong China; 5 Department of Traditional Chinese Medicine, Shandong Provincial Qianfoshan Hospital, The First Affiliated Hospital of Shandong First Medical University, No. 16766 Jingshi Road, Jinan, 250014 Shandong China; 6 Department of Gynecology, The Affiliated Hospital of Shandong University of Traditional Chinese Medicine, No. 16369 Jingshi Road, Jinan, 250014 Shandong China; 7 Department of Traditional Chinese Medicine, Shandong Provincial Hospital Affiliated to Shandong First Medical University, No. 324 Jingwu Road, Jinan, 250021 Shandong China

**Keywords:** Adenomyosis, Single-cell RNA sequencing, Malignant tumour characteristic, Epithelial-endothelial transition, Vasculogenic mimicry

## Abstract

**Background:**

Adenomyosis (AM) is a common benign chronic gynaecological disorder; however, the precise pathogenesis of adenomyosis is still poorly understood. Single-cell RNA sequencing (scRNA-seq) can uncover rare subpopulations, explore genetic and functional heterogeneity, and reveal the uniqueness of each cell. It provides us a new approach to reveal biological issues from a more detailed and microscopic perspective. Here, we utilize this revolutionary technology to identify the changes of gene expression patterns between ectopic lesions and the eutopic endometrium at the single-cell level and explore a potential novel pathogenesis of AM.

**Methods:**

A control endometrium (sample with leiomyoma excluding endometrial disorders, n = 1), eutopic endometrium and ectopic lesion (from a patient with adenomyosis, n = 1) samples were analysed by scRNA-seq, and additional leiomyoma (n = 3) and adenomyosis (n = 3) samples were used to confirm colocalization and vasculogenic mimicry (VM) formation. Protein colocalization was visualized by immunofluorescence, and CD34-periodic acid-Schiff (PAS) double staining was used to assess the formation of VM.

**Results:**

The scRNA-seq results suggest that cancer-, cell motility- and inflammation- (CMI) associated terms, cell proliferation and angiogenesis play important roles in the progression of AM. Moreover, the colocalization of EPCAM and PECAM1 increased significantly in the ectopic endometrium group (P < 0.05), cell subpopulation with high copy number variation (CNV) levels possessing tumour-like features existed in the ectopic lesion sample, and VNN1- and EPCAM-positive cell subcluster displayed active cell motility in endometrial epithelial cells. Furthermore, during the transformation of epithelial cells to endothelial cells, we observed the significant accumulation of VM formation (positively stained with PAS but not CD34, P < 0.05) in ectopic lesions.

**Conclusions:**

In the present study, our results support the theory of adenomyosis derived from the invasion and migration of the endometrium. Moreover, cell subcluster with high CNV level and tumour-associated characteristics is identified. Furthermore, epithelial-endothelial transition (EET) and the formation of VM in tumours, the latter of which facilitates the blood supply and plays an important role in maintaining cell growth, were also confirmed to occur in AM. These results indicated that the inhibition of EET and VM formation may be a potential strategy for AM management.

**Supplementary Information:**

The online version contains supplementary material available at 10.1186/s13578-021-00562-z.

## Background

Adenomyosis (AM) is a common uterine disorder with clinical manifestations such as abnormal uterine bleeding, menorrhagia, dysmenorrhoea, dyspareunia and infertility, and it is defined by the heterotopic growth of endometrial glands and stroma within the myometrium [1–3]. The symptoms of AM affect the quality of life for women, especially for women with a desire to have children. It is believed that a mean proportion of 20–35% of women worldwide suffer from AM [4]. Furthermore, there is evidence showing that the incidence rate has presented an increasing trend because of the optimization of diagnostic imaging techniques such as transvaginal ultrasound scan (TVUS) and magnetic resonance imaging (MRI), and the increased proportion of young women diagnosed is remarkable [5, 6]. Nevertheless, the clinical diagnosis of AM is still a challenging problem because its symptoms are nonspecific. In addition, its imaging characteristics are similar to those of other gynaecological diseases (such as leiomyoma, endometrial polyps and endometriosis) [7]. Moreover, the diagnostic criteria for imaging have not been unified.

Effective clinical treatment of AM is also difficult. Currently, various clinical and pharmacological studies have been performed to find an effective therapeutic compound for AM [8]. However, there are no well-studied drugs for AM patients. Hormone drugs such as gonadotrophin-releasing hormone agonist (GnRHa), Mirena and progestins have been used to relieve the symptoms of AM [9, 10]. Unfortunately, these hormonal therapies do not perform well in the long term, and they are usually accompanied by side effects such as nausea, vomiting, acne, irregular vaginal bleeding and osteoporosis [8]. In addition, those patients whose symptoms do not improve after pharmacotherapy or even relapse after drug withdrawal undergo invasive surgeries, which are not suitable for women who desire to preserve their fertility [11]. The limitations of the present clinical diagnosis and conservative treatments, to a considerable extent, are attributed to the poor understanding of the pathogenesis and aetiology of AM. Thus, it is necessary to reveal the potential molecular mechanism of the disorder, which may provide a new strategy for clinical diagnosis and medication.

There are many hypotheses about the pathogenesis of AM, and two main theories are used to elucidate its origin. The first and most well-known theory is that AM derives from the invagination of the endometrial basalis, which is based on the enhancement of invasion and migration ability of endometrium, myometrial weakness, altered immunological activity at the endometrial-myometrial interface and abnormal secretion of hormones [12, 13]. This process is similar to the invasion and metastasis of malignant tumours. In addition, it was proposed that AM is associated with embryological dislocation of multiple Müllerian residues and differentiation of endometrial stem/progenitor cells [2, 12]. Nevertheless, the pathogenesis of AM is still elusive, and none of the above theories could explain all phenotypes of the disorder; thus, more evidences are needed to verify these hypotheses. In addition, the application of new technology may provide a better strategy for a better interpretation of its pathogenesis.

At present, as a revolutionary technology, single-cell RNA sequencing (scRNA-seq) can uncover new cell types and rare subpopulations, explore genetic and functional heterogeneity, and determine the mechanisms of gene regulation and random allele expression to reveal the uniqueness of each cell [14–18]. Therefore, this technology may help us solve difficult biological questions from a more detailed and microscopic perspective [16]. Moreover, scRNA-seq has been used in the research of reproductive diseases. Ferrero *et el.* reported the role that endometriosis played in oocytes from endometriotic ovaries by using scRNA-seq technology to calculate the global transcriptome behaviour [19]. Evidence from Saatcioglu et al. proposed that Misr2 + cells could affect uterine hypoplasia and cause complete infertility through scRNA-seq [20]. Therefore, the scRNA-seq technique may be beneficial for researchers to identify the pathogenesis or diagnostic indicators of AM. We assumed that eutopic and ectopic endometrial cells from AM patients have different gene expressions, which was likely related to dysmenorrhoea, irregular uterine bleeding, endometrial receptivity and other clinical manifestations of AM. In the present study, we used scRNA-seq to detect the control endometrium from hysteromyoma patient, eutopic endometrium and ectopic endometrium of AM patient with the aim of exploring new information about the pathogenesis of AM.

## Results

### Single-cell analysis of the eutopic endometrium and ectopic lesions of AM

**Fig. 1:**
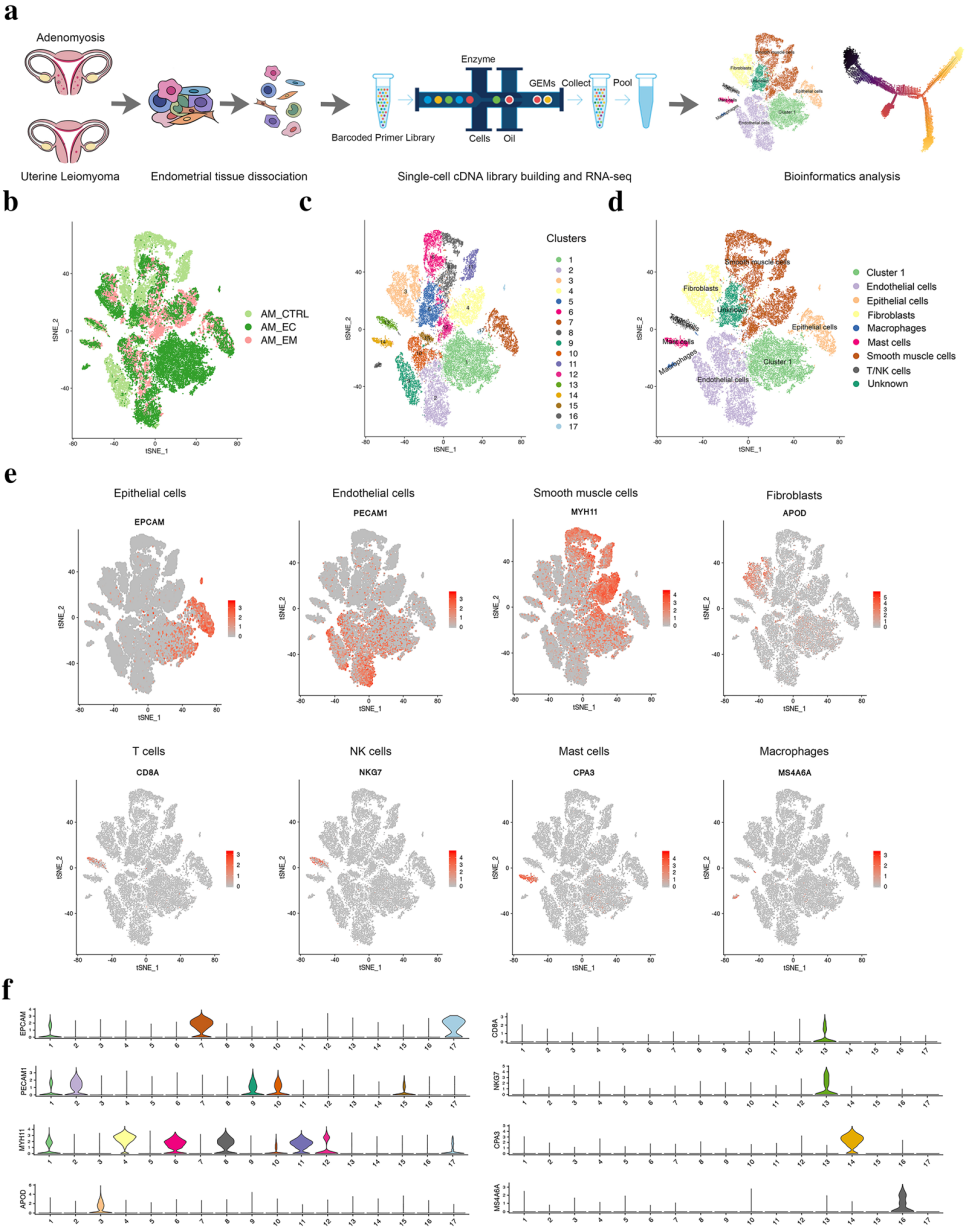
Identification of endometrium populations with single-cell transcriptomic analysis. **a** The workflow shows the collection and processing of obtained endometrium samples of AM and control hysteromyoma for scRNA-seq. *t*-distributed stochastic neighbour embedding (*t*-SNE) visualization of all cells displayed with different colours for samples (**b**), clusters (**c**) and cell types (**d**). **e** Representative markers of different cell types are shown. **f** For violin plots, x-axes stand for numbers of clusters, y-axes stand for relative expression level of corresponding genes, colors correspond to the colors of each cluster in **c**, and shapes represent the number of cells corresponding to the relative expression level in y-axis

To understand the potential molecular basis of the development of AM, samples were obtained from patients and analysed by scRNA-seq. To this end, eutopic endometrium (AM_EM group) and ectopic endometrium (AM_EC group) samples were obtained from total hysterectomies of patients with AM, and endometrial tissue from patients with hysteromyoma served as the control (AM_CTRL group). Hematoxylin–eosin (HE) staining was used to detect the histological characteristics, and we found that the gland invaded the muscle layer of the AM_EC group (Additional file 1: Figure S1A), which was consistent with the characteristics of AM. To explore cell diversity, we generated scRNA-seq profiles from the 3 groups mentioned above (Fig. 1a). Our scRNA-seq data were analysed with strict quality control (QC) criteria according to the method (Additional file 1: Figure S1B-G). A total of 42,292 cells, including cells from the AM_CTRL, AM_EM and AM_EC groups, was sequenced, of which 36,781 cells were used for a following analysis after QC (Additional file 1: Figure S1G). Cell doublets were detected by Scrublet, the proportion of mitochondrial genes, the number of genes expressed, and the number of unique molecular identifiers (UMIs) in each cell are shown in the violin plots (Additional file 1: Figure S1C-F). After the completion of QC, *t*-distributed stochastic neighbour embedding (*t*-SNE) analysis was conducted, and 17 cell clusters were obtained (Fig. 1b, c and Additional file 1: Figure S2A). The top ten genes expressed in each cluster are shown in the heatmap (Additional file 1: Figure S2B). The 17 clusters were divided into 7 different cell types according to cell type-specific gene expression. In addition, there was also an unidentified cluster (named unknown) and an unnamed cluster (cluster 1) (Fig. 1d and Additional file 1: Figure S2C). In the process of cell type identification, widely recognized cell markers were used, such as EPCAM for epithelial cells, PECAM1 for endothelial cells, and APOD for fibroblasts (Fig. 1e, f). Various additional cell markers were also used to identify cell types, such as CNN1 for smooth muscle cells, MS4A7 for macrophages, and CD3E for T cells (Additional file 1: Figure S2D,E) (representative marker reference from: http://biocc.hrbmu.edu.cn/CellMarker/ and https://panglaodb.se/search.html). Cluster 1 was a special cell group, and the results of cell type identification showed that it expressed markers in different cells, including epithelial cells and endothelial cells (Fig. 1e, f). Its characteristics will be deeply studied.

### Cluster 1 possessed colocalization of epithelial and endothelial markers and high copy number variation (CNV) levels

**Fig. 2:**
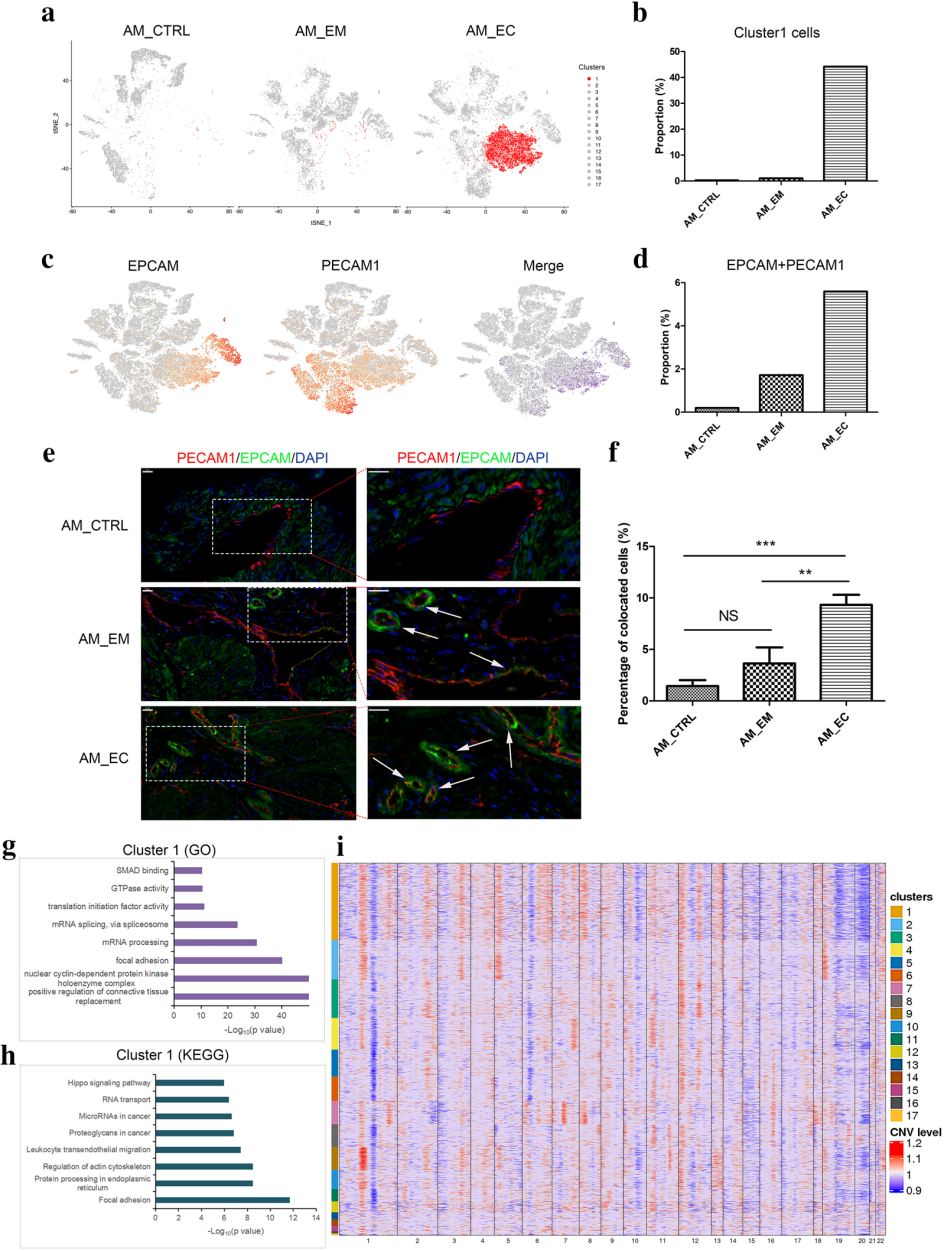
Colocalization of EPCAM and PECAM1 in cluster 1 characterized by high CNV levels. The distribution of cluster 1 in the *t*-SNE map (**a**) and proportion (**b**) of the three groups are shown. **c** EPCAM-positive, PECAM1-positive and double EPCAM- and PECAM1-positive cells are displayed in the *t*-SNE map. **d** The histogram shows the proportion of EPCAM- and PECAM1-positive cells in the three samples. **e** PECAM1 (red), EPCAM (green) and nuclei (blue) were stained, and the white arrows show the colocalization of EPCAM and PECAM1, scale bars = 20 μm. **f** Histogram indicates the percentage of colocated cells in different groups; ns represents not significant, **P < 0.01, ***P < 0.001. The enriched GO (**g**) and KEGG (**h**) terms of cluster 1 are shown. **i** The heatmap represents the large-scale CNV levels of cluster 1 to cluster 17 across chromosomes 1 to 22; red indicates a high CNV level, and blue indicates a low CNV level. GO, Gene Ontology; KEGG, Kyoto Encyclopedia of Genes and Genomes; CNV, copy number variation

By comparing the distribution of cluster 1 in the three groups of samples, it was found that the proportion of cluster 1 in the AM_CTRL, AM_EM and AM_EC groups had an increasing trend (Fig. 2a, b), especially in the AM_EC sample. Moreover, the tissues of the AM_EC samples were taken from ectopic lesions of AM, indicating that cluster 1 may be closely related to AM. Markers of different cell types were expressed in cluster 1, particularly according to the distribution of EPCAM and endothelial cell marker (PECAM1) in the *t*-SNE map, suggesting that there was colocalization of the two markers in some of the cells belonging to cluster 1. Moreover, the number of colocalized cells in the AM_EC group was greater than those in the AM_CTRL and AM_EM groups (Fig. 2c, d). In addition, the colocalization of CDH1 and VWF, KRT7 and CDH5 also indicated the coexpression of epithelial and endothelial markers in cluster 1 (Additional file 1: Figure S3A, B). The results of doublet cell test showed that very low fraction of doublets were observed in the overall scRNA-seq data and Cluster 1 (Additional file 1: Figure S1C and Additional file 2: Table S1). This indicated that colocalization of the two types of cell markers might do exist, rather than mainly caused by doublet cells. Furthermore, the immunofluorescence results then revealed the colocalization of EPCAM and PECAM1 in the corresponding tissues of the AM_EM and AM_EC samples (Fig. 2e), and the colocalized cells accumulated significantly in AM_EC group (Fig. 2f). In addition, the proportion of cells that colocalized with the two markers of the verifiable AM_EC (V) group was significantly higher than that of the AM_EM (V) group and AM_CTRL (V) group (Additional file 1: Figure S3C, P < 0.05), which was consistent with the trend of colocalization in the *t*-SNE map (Fig. 2a, b). These results indicated that cluster 1 was a unique cluster, which was worth studying to determine its genetic background.

The *t*-SNE map classified cells based on the similarity of gene transcription, and there was colocalization of the epithelial and endothelial markers, which indicated that the cell cluster might possess malignant characteristics. For the top 500 genes expressed in cluster 1, Gene Ontology (GO) and Kyoto Encyclopedia of Genes and Genomes (KEGG) pathway analyses revealed that cancer-, cell motility- and inflammation-associated terms (CMI terms) were enriched in cluster 1 (Fig. 2g, h). Moreover, compared with other clusters (clusters 2–17), cluster 1 was enriched for CMI- and cell growth-associated terms (Additional file 1: Figure S3D, E). Compared with epithelial cell populations (clusters 7 and 17), cluster 1 showed the accumulation of angiogenesis-, cell growth-, cancer- and motility-related terms (Additional file 1: Figure S3F, G). Hence, chromosomal CNV analysis was carried out according to the average expression patterns across intervals of the genome. The results show that cluster 1 had a high level of CNV (Fig. 2i).

Taken together, our results show that there were colocalization markers of epithelial cells and endothelial cells in cluster 1, and it possessed a high CNV level. Furthermore, the terms of CMI, angiogenesis and cell growth, which contribute to tumour progression, were enriched in cluster 1. The above results indicate that malignant cell populations were present in the ectopic endometrium, which was consistent with AM possessing tumour-like characteristics [21].

### Gene expression pattern analyses in AM_CTRL, AM_EM and AM_EC samples.

The tissues were derived from eutopic endometrium and ectopic lesions, which prompted us to analyse the gene expression patterns of the AM_EC and AM_EM groups. Compared with the AM_EM group, 535 genes were differentially expressed in the AM_EC group, among which 383 genes were upregulated and 152 genes were downregulated (Additional file 3: Table S2). GO analysis was performed on the upregulated genes, and the gene functions were mainly enriched for terms involved in angiogenesis, cell motility, and cell growth and survival, including positive regulation of angiogenesis, positive regulation of cell migration, growth factor binding and negative regulation of apoptotic process (Additional file 1: Figure S4A). The AM_EM group was compared with the AM_CTRL group, and GO analysis showed that the functions of upregulated genes were also enriched in cell motility- and cell growth-associated terms, such as structural constituent of cytoskeleton, Rho GTPase-binding and growth factor-binding (Additional file 1: Figure S4B). KEGG analysis showed that compared with those of the AM_EM group, the enrichment items of the AM_EC group were mainly concentrated in CMI- and cell proliferation-related terms, such as pathways in cancer, focal adhesion, the NF-κB signalling pathway and the PI3K-Akt signalling pathway (Additional file 1: Figure S4C). Furthermore, cell motility-, inflammation-, and cell proliferation-related terms, such as focal adhesion, leukocyte transendothelial migration and the MAPK signalling pathway, were also enriched in the AM_EM group compared with the AM_CTRL group (Additional file 1: Figure S4D). There were 43 differentially expressed genes (DEGs) coexisting in AM_EM versus AM_CTRL (196 DEGs) and AM_EC versus AM_EM (148 DEGs) (Additional file 1: Figure S4E and Additional file 4: Table S3). The functions of coexisting DEGs were mainly focused on angiogenesis, cell mobility-related cytoskeleton regulation and chemotaxis (Additional file 1: Figure S4F). Taken together, the results reveal that the disease progression of AM was closely related to CMI terms. Furthermore, a series of GO and KEGG terms enriched in ectopic lesions partially appeared in the eutopic endometrium of AM patients.

**Fig. 3:**
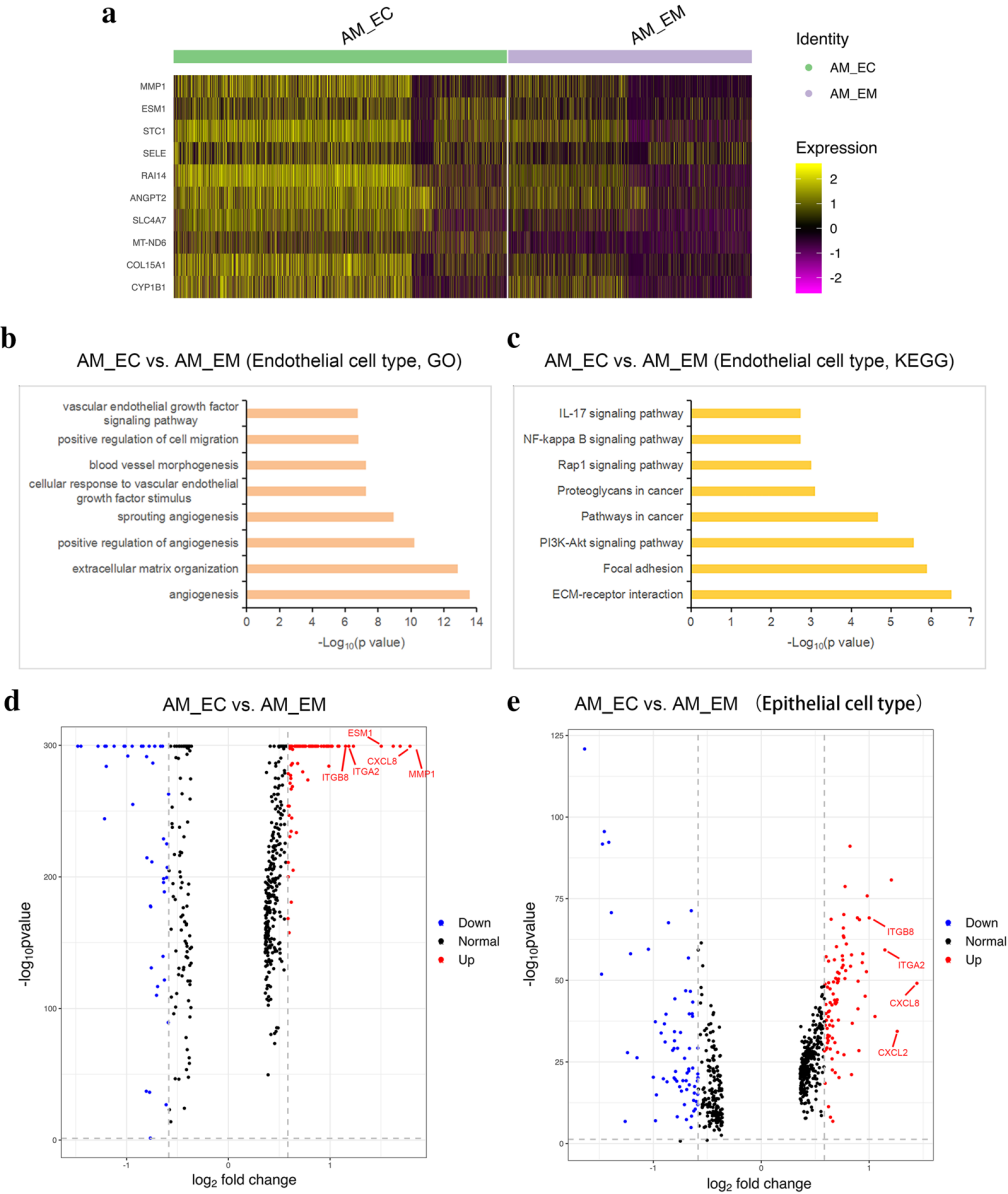
Gene expression pattern analyses in the AM_EM and AM_EC samples. **a** A heatmap revealed the expression levels of specific genes in the AM_EC and AM_EM groups. Yellow indicates a high expression level, and violet indicates a low expression level. Enriched GO (**b**) and KEGG (**c**) terms of endothelial cell populations in the AM_EC group compared with the AM_EM group are shown. **d** Volcano plot depicting gene expression differences between the AM_EC group versus the AM_EM group. Representative upregulated genes are shown. **e** Volcano plots indicate gene expression differences in epithelial cells between the AM_EC group and the AM_EM group, and representative upregulated genes are shown. *GO* Gene Ontology, *KEGG* Kyoto Encyclopedia of Genes and Genomes

A heatmap showed that genes related to angiogenesis in the AM_EC group were upregulated compared with those in the AM_EM group, including MMP1 [22], ESM1 [23], ANGPT2 [24] and CYP1B1 [25] (Fig. 3a). GO and KEGG analyses in endothelial cells indicated that multiple angiogenesis-associated terms were accumulated in the AM_EC group versus the AM_EM group, in addition, CMI- and proliferation-related terms, such as pathways in cancer, positive regulation of cell migration, NF-κB signalling pathway and PI3K-Akt signalling pathway, were also enriched in the comparison (Fig. 3b, c). These results suggest that endothelial cells in the AM_EC group possessed some malignant characteristics and that the angiogenic ability was enhanced.

The DEGs (p value < 0.05, fold change > 1.5) in AM_EC versus AM_EM are presented as volcano maps, and analysis of the upregulated genes with log_2_ fold change > 1 showed that the functions of a series of genes were related to angiogenesis (such as MMP1 and ESM1), inflammation (such as CXCL8 [26]) and cell motility (such as ITGA2 [27] and ITGB8 [28]) (Fig. 3d). It has been reported that the pathogenesis of AM is closely related to the enhancement of invasion and migration of endometrial cells and epithelial-mesenchymal transition (EMT) [29, 30]; therefore, the cell motility of epithelial cell clusters became our focus. The DEGs of epithelial cells in the AM_EC versus AM_EM groups were analysed, and ITGA2, ITGB8 and CXCL8 were also identified in the upregulated genes with a log_2_ fold change > 1 (Fig. 3e). These results suggest that the enhancement of cell motility and migration in the AM_EC group compared with those in the AM_EM group may be related to the epithelial cell group, which is worthy of further study.

### Distinct subclusters in the epithelial cell group.

**Fig. 4:**
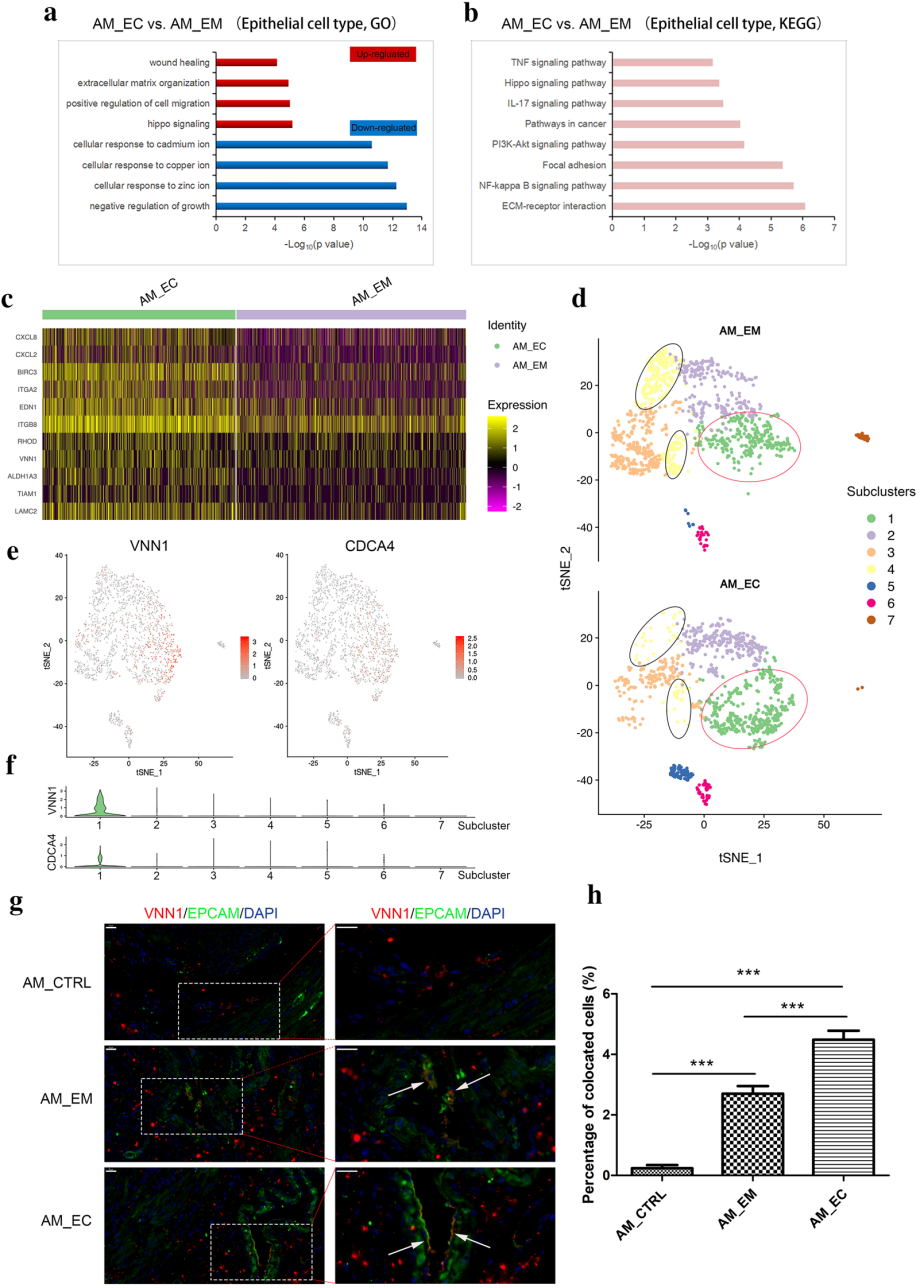
Gene expression pattern and characteristics of the epithelial cell population. Representative GO (**a**) and KEGG (**b**) terms of epithelial cell populations in the AM_EC group compared with the AM_EM group are shown. **c** A heatmap revealed the expression levels of the indicated genes in the AM_EC and AM_EM groups. Yellow indicates a high expression level, and violet indicates a low expression level. **d** The *t*-SNE map indicated subclusters of epithelial cells in the AM_EM and AM_EC groups, in which subcluster 1 is circled in red and subcluster 4 is circled in black. The *t*-SNE map (**e**) and violin plots (**f**) show the distribution of VNN1 and CDCA4 in each subcluster. **g** VNN1 (red), EPCAM (green) and nuclei (blue) were stained, and the white arrows show the colocalization of VNN1 and EPCAM, scale bars = 20 μm. **h** Histogram indicates the percentage of colocated cells in different groups; ***P < 0.001. GO, Gene Ontology; KEGG, Kyoto Encyclopedia of Genes and Genomes

In the epithelial cell population, compared with those in the AM_EM group, the upregulated genes (fold change > 1.5) in the AM_EC group were evaluated by GO analysis, and the main functions of these genes were enriched in the items related to cell motility, including positive regulation of cell migration, extracellular matrix organization, wound healing, etc. (Fig. 4a). KEGG analysis showed that in addition to the items related to cell motility, inflammatory-related pathways, such as the NF-κB signalling pathway, IL-17 signalling pathway and TNF signalling pathway, were also enriched among the upregulated DEGs (Fig. 4b). Moreover, the genes that promote cell mobility, including ITGA2, ITGB8, RHOD [31], VNN1 [32, 33], ALDH1A3 [34], and TIAM1 [35], were highly expressed in the AM_EC group (Fig. 4c). These results indicate that the epithelial cells derived from AM exhibited high motility.

Seven subclusters were identified in epithelial cells based on *t*-SNE analysis (Additional file 1: Figure S5A); moreover, the proportion of subcluster 1 in the AM_EC group was higher than that in the AM_EM group, while the proportion of subcluster 4 followed the opposite trend (Fig. 4d and Additional file 1: Figure S5B, C). Through the heatmap visualization of gene expression, we found that each subcluster had different gene expression characteristics. Moreover, genes related to cell migration, such as RHOD, TIAM1, VNN1, and ALDH1A3, were mainly enriched in subcluster 1 (Additional file 1: Figure S5D). This indicates that subcluster 1 may be a group of cells with a high migratory ability. Since the pathogenesis of AM is related to endometrial invasion, migration and EMT, functional enrichment analysis was conducted for subcluster 1, and the results suggest that the specific functions of subcluster 1 mainly focus on cell migration and related cytoskeleton regulation (Additional file 1: Figure S5E), which is consistent with the enrichment of migration-related genes in subcluster 1. The proportion of subcluster 1 cells in the AM_EC sample was higher than that in the AM_EM sample (Additional file 1: Figure S5B); thus, more cells with an active migratory ability accumulated in the AM_EC group. KEGG analysis in subcluster 1 revealed that this cell group was mainly enriched for cancer-associated terms (Additional file 1: Figure S5F), which was identical to the report that AM possessed malignant tumour features. VNN1 participated in the regulation of cell migration and specifically existed in subcluster 1 (Fig. 4e, f); therefore, EPCAM, as a marker gene of epithelial cells, was costained with VNN1 to identify the existence of this cell group in tissue samples. EPCAM and VNN1 colocalization emerged significantly in the AM_EM and AM_EC groups (Fig. 4g, h). These results suggest that there was a group of cells with high migratory ability in the epithelial cells of AM, which contributes to the progression of the disorder.

The proportion of subcluster 4 in the AM_EM group was higher than that in the AM_EC group (Additional file 1: Figure S5C), and the functions of the subcluster were mainly focused on the regulation of cell survival, transcription and mRNA processing (Additional file 1: Figure S5G). KEGG analysis showed that inflammation-related pathways and antigen processing and presentation were enriched in this subcluster (Additional file 1: Figure S5H); thus, subcluster 4 might be involved in inflammation. In general, these results indicate that functions related to cell migration, tumour, cell survival and inflammation in AM had begun to accumulate in epithelial cells; however, the proportion of corresponding subclusters was different between the AM_EC and AM_EM groups.

### Pseudotime trajectory analysis of the epithelial cell, cluster 1 and endothelial cell groups reveals epithelial transition to endothelial cells

In view of the existence of epithelial cells and endothelial cells in the identification of cell types, cluster 1 was sandwiched in the middle next to the epithelial cells and endothelial cell groups in the *t*-SNE map, and there was colocalization of epithelial cells and endothelial cell markers in cluster 1. In addition, it has been reported that epithelial-endothelial transition (EET) may occur in the process of tumour progression, which is considered a subtype of EMT [36]. Accumulating evidence indicates that there may be a transition between epithelial cells and endothelial cells in AM; therefore, pseudotime trajectory analysis was conducted in the epithelial cell, cluster 1 and endothelial cell groups.

**Fig. 5:**
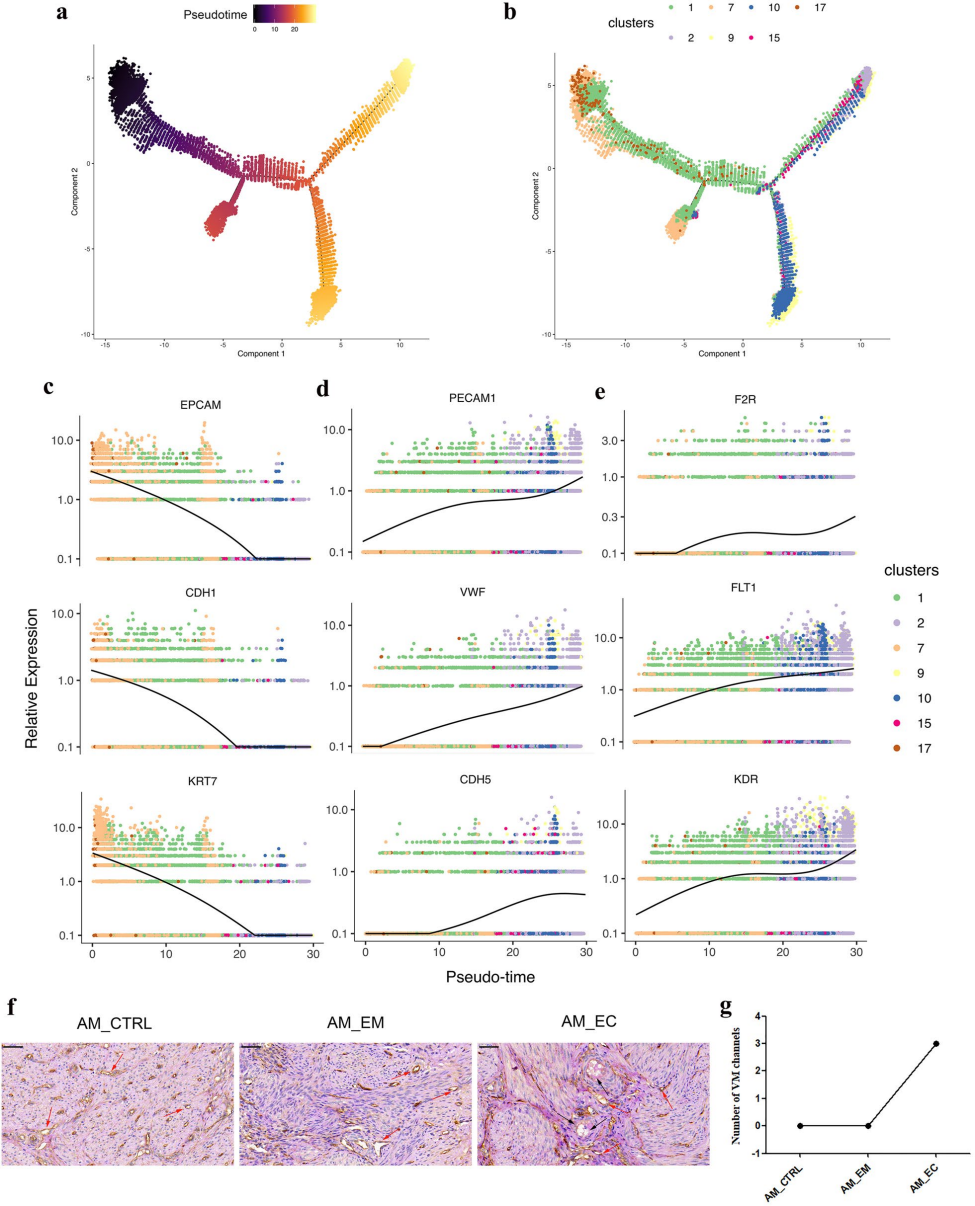
Reconstruction of the EET trajectory in a pseudotime manner. **a** Trajectory reconstruction of all single cells from epithelial cells to endothelial cells and **b** the distribution of clusters on the trajectory are shown. Gene expression dynamics of representative genes of epithelial cell markers (**c**), endothelial cell markers (**d**) and angiogenesis-related markers (**e**) are displayed. **f** CD34-PAS double-staining demonstrated VM channels in AM_CTRL, AM_EM and AM_EC samples. VM channels (black arrows) were positive for PAS staining but negative for CD34 staining (red arrows represent channels positive for CD34), scale bars = 50 μm. **g** Line chart indicates the numbers of VM channels in the different groups. *VM* vasculogenic mimicry, *PAS* Periodic Acid-Schiff

To reveal this relationship in transcriptology and to study the genes that regulate this process, cells were arranged in a pseudotime manner with a pedigree reconstruction algorithm for biological processes based on transcriptional similarity [37, 38]. We found that the cells in epithelial cell, cluster 1 and endothelial cell groups formed a continuous trajectory (Fig. 5a, b), and the groups and cell types on trajectory was indicated (Additional file 1: Figure S6A, B). Furthermore, markers of epithelial cells (EPCAM, CDH1 and KRT7) and endothelial cells (PECAM1, VWF and CDH5) were mapped on the trajectory (Additional file 1: Figure S6C, D). The above results suggest that epithelial cells transited to endothelial cells according to the continuous pseudotime trajectory, and only AM_EC group, but not AM_CTRL and AM_EM groups, could form continuous trajectory. The epithelial cell type and its markers were mainly located on the left side of the pseudotime trajectory, and the endothelial cell type and its markers were mainly distributed on the right side of the pseudotime trajectory, while cluster 1 cells were distributed over the entire trajectory (Additional file 1: Figure S6B-D). The distribution of cluster 1 was partly related to its characteristics, including epithelial cells, endothelial cells and epithelial and endothelial marker coexpression cells.

Gene expression dynamics showed that the relative expression levels of epithelial cell markers (EPCAM, CDH1 and KRT7) decreased as pseudotime progressed (Fig. 5c); however, that of endothelial cell markers (PECAM1, VWF and CDH5) increased (Fig. 5d). Moreover, the relative expression levels of angiogenesis-related and vasculogenic mimicry (VM) formation-associated markers (F2R, FLT1 and KDR) [36] were also upregulated (Fig. 5e). This indicates that angiogenesis is enhanced with the transformation of epithelial cells into endothelial cells. Since EET could produce the endothelial-like phenotype of tumour cells and promote the formation of VM to enhance the blood supply [36, 39], VM formation was assayed by CD34-periodic acid-Schiff (PAS) double staining. The results show that the number of VM formations in the AM_EC group was increased in the sequencing samples (Fig. 5f, g). Moreover, in the verified samples, the number of VM in AM_EC (V) was significantly increased compared with that in AM_CTRL (V) and AM_EM (V) (P < 0.05). Nevertheless, there was no significant difference between AM_CTRL (V) and AM_EM (V) (Additional file 1: Figure S6E, F). Taken together, the epithelial cell, cluster 1 and endothelial cell groups exhibited EET, with increases in angiogenic factor expression levels and VM formation, which were beneficial to the blood supply.

## Discussion

Adenomyosis is a common benign chronic gynaecological disorder; however, it also displays some malignant tumour features, including invasion, migration and abnormal proliferation [21]. The endometrium directly invaded the myometrium, and misplaced pluripotent Müllerian remnants were proposed to cause the pathogenesis of AM [6]. Moreover, inflammatory factors, such as LOX5 and COX2, are related to dysmenorrhoea and menstruation [40], and it has also been reported that angiogenesis participates in the pathophysiology of abnormal uterine bleeding and subfertility in AM [3]. Nevertheless, the precise pathogenesis of AM is still poorly understood. In the present study, we explored the differences of gene expression patterns at single-cell level between ectopic and eutopic endometrium of AM using scRNA-seq technology, and the potential important mechanisms that affect the progression were discussed.

Although there was one sample in each of the AM_CTRL, AM_EM and AM_EC groups, the results of scRNA-seq were based on the duplication of multiple cells of the same cell type. In order to increase the credibility of our results, additional leiomyoma (n = 3) and adenomyosis (n = 3) samples were used to confirm EPCAM and PECAM1 colocalization and VM formation.

Compared with the AM_EM sample, the AM_EC sample was enriched for cell motility and inflammation-related terms according to the GO and KEGG results, and the volcano map results showed that associated gene transcription, including that of ITGA2 [27], ITGB8 [41] and CXCL8 [42], was upregulated. Since the pathogenesis of AM is closely related to the functional changes in the endometrium [43], we identified the functional changes in endometrial epithelial cells in the AM_EC and AM_EM groups and found that several identical items related to cell movement and inflammation, including positive regulation of cell migration and the IL-17 signalling pathway, appeared in the comparison of the AM_EC versus AM_EM groups in epithelial cells. Moreover, ITGA2, ITGB8 and CXCL8 also emerged in the top 10 upregulated genes in the comparison between the two groups (Additional file 5: Table S4). These results further indicate that epithelial cell groups might play an essential role in the progression of AM. The cell motility, cell proliferation, angiogenesis and inflammation terms were enriched in both AM_EC versus AM_EM and AM_EM versus AM_CTRL, and forty-three DEGs coexisted in AM_EM versus AM_CTRL and AM_EC versus AM_EM. The coexisting DEGs mainly functioned in angiogenesis and cell mobility-related cytoskeleton regulation and chemotaxis. However, cancer-associated items, including pathways and miRNAs in cancer, emerged in AM_EC versus AM_EM. These findings suggest that some pathological feature changes in AM had already appeared in the eutopic endometrium, and some of these alterations were identical to the reporting that AM possessed tumour-like characteristics [21].

There were epithelial cells in the *t*-SNE map of the AM_EC and AM_EM groups, but the number of epithelial cells in the corresponding position of the AM_CTRL group was very low, which was mainly attributed to the endometrium being obviously thin, as determined by postoperative identification in the AM_CTRL group. In the follow-up comparison of the epithelial cell groups, first because of the small number of epithelial cells in the AM_CTRL group and second because of the potential individual differences, only the AM_EC and AM_EM samples were compared, and the results show that the function of upregulated genes (p value < 0.05, fold change > 1.5) mainly focused on CMI- related terms. These results are consistent with the malignant tumour features of AM, including invasion and migration [21, 29], and a subcluster1 with active motility is identified in epithelial cells. Thus, the present study support the theory of AM derived from the invasion and migration of endometrium.

Lang et al. considered that compared with the normal endometrium, the eutopic endometrium of patients with endometriosis exhibited fundamental aberrant changes [44], which may also be applicable to AM. Our results suggest that cell motility-, inflammation-, and cell proliferation-associated terms were not only enriched in the functional analysis of the AM_EC versus AM_EM group but also emerged in the analysis of the AM_EM versus AM_CTRL group. Furthermore, in the analysis of the epithelial cell population, we found that the high-motility cell group was located in subcluster 1, and the inflammation- and antigen processing- and presentation-related cells were located in subcluster 4; however, the existence of these two subclusters in the AM_EC and AM_EM samples was confirmed by a *t*-SNE map. As a kind of endometriosis, our results show that cell motility, inflammation and other functions related to the pathogenesis of AM have accumulated in the eutopic endometrium of patients, which also supports “eutopic endometrium determinism” [45].

The number of cells in cluster 1 in the AM_CTRL, AM_EM and AM_EC samples displayed an increasing trend. AM_EC was taken from the cells in the lesion area of AM, and the proportion of cluster 1 in AM_EC increased sharply, suggesting that it was closely associated with AM. The CNV level of cluster 1 was high, and immunofluorescence showed the colocalization of epithelial and endothelial cell markers, which indicated that it possessed malignant characteristics, and was identical to the reported tumour-like features of AM. Although there are multiple biological replicates of the same cell cluster in single-cell sequencing, due to individual differences, additional samples from six participants were used for validation, and significant EPCAM and PECAM1 colocalization was found in the AM_EC (V) group (P < 0.05). However, it is precisely because of its malignant characteristics that the top ten markers in the gene_diff item of this cluster were not only distributed in this cell population, so it could not be determined by immunofluorescence or immunohistochemistry that this cell group exists in only cluster 1.

CMI-related terms recurred in multiple comparisons between groups and the enrichment of upregulated DEGs, which suggested that canceration, cell motility and inflammation may play an important role in the process of AM. The study of AM suggested that inflammation accumulated in the eutopic endometrium sample compared with in the control sample [40, 46], which was supported by the results of the comparison between the AM_EM and AM_CTRL groups in the present study (Additional file 1: Figure S4B, D). In addition, the inflammatory terms were further enriched in the AM_EC group; however, the proportion of inflammation-related subcluster 4 cells in the epithelial cell group was higher in the AM_EM sample than in the AM_EC sample, which may facilitate disease progression because inflammation can promote cell migration and invasion [47]. Moreover, invasion and migration play an important role in the progression of AM, which has been supported by relevant reports [48].

Studies have shown that when the tumour reaches 1–2 mm in diameter, angiogenesis is essential to maintain its growth [36]. However, angiogenesis is complex, and tumours that cannot form blood vessels completely rely on host endothelial cells. Other angiogenesis pathways have been proposed, including the formation of vascular-like structures, such as VM [39]. EET is considered a subtype of EMT, which can produce the endothelial-like phenotype of tumour cells, and VM is formed through this endothelial-like phenotype to allow a sufficient blood supply [36, 39]. In this study, we found that cluster 1 has tumour-like characteristics, and the cluster possessed epithelial cells, epithelial cell marker and endothelial cell marker coexpression cells and endothelial cells. In addition, pseudotime analysis also showed that EET occurred in epithelial cells, cluster 1 and endothelial cells. The results of CD34 and PAS costaining suggest that VM formation increased significantly in the confirmatory AM_EC (V) samples (P < 0.05). The present study indicates that EET and the formation of VM present in tumours, which facilitates the blood supply and plays an important role in maintaining cell growth, also occur in AM.

## Conclusions

AM threatens women's health and is presenting a younger patient trend, and no curative drugs are currently available; nevertheless, the pathogenesis of the disease is poorly understood. In the present study, our scRNA-seq results support the theory of AM derived from the invasion and migration of endometrium and suggest that CMI terms, cell proliferation and angiogenesis play important roles in the progression of AM. In addition, there were cell subpopulations with malignant tumour characteristics in the ectopic lesion sample, and the transition from epithelial cells to endothelial cells in the ectopic lesions of AM was occurred with the formation of VM. Thus, EET and VM formation may be a novel pathogenesis of AM and inhibition of EET and VM formation may be a potential strategy for AM management.

## Methods

### AM patient samples

Local institutional review board approved the project (the Affiliated Hospital of Shandong University of Traditional Chinese Medicine, Jinan, China), and patients diagnosed with hysteromyoma and AM were signed informed consent. Fresh AM tissues were obtained by laparoscopic surgery from a 46-year-old female AM patient. The operation was carried out under the supervision of experienced gynaecological surgery experts, and the diagnosis was subsequently confirmed by histopathological examination. Eutopic endometrium was marked as AM_EM, and ectopic endometrium was marked as AM_EC. The control sample (AM_CTRL) in the project was obtained from a 50-year-old female patient with hysteromyoma through laparoscope, and endometriosis and AM disease were excluded. The samples used in the verification experiment were obtained according to the above methods. Information about the patients is listed in Additional file 6: Table S5.

### Preparation of single-cell suspensions

Samples of the eutopic endometrium and ectopic lesions from patient with AM and eutopic endometrium from patient with hysteromyoma were isolated on the same day and transported rapidly to the research facility. Then, each sample was chopped into cubes of no more than 1 mm under the condition of 4℃, followed by digestion with collagenase, shaking evenly every 5 min. Next, the supernatant was removed after centrifugation at 300 relative centrifugal force (RCF) for 30 s at room temperature (RT), without disturbing the cell particles. BSA (400 µg/ml) prepared with 1 × PBS free with calcium and magnesium was added, after which the samples were centrifuged at 300 RCF for 5 min at RT, and the cell pellets were resuspended in 1 ml red blood cell lysate buffer, followed by incubation at 4℃ for 10 min. After red blood cell lysis, the samples were resuspended in 1 ml of BSA. Finally, the processed samples were filtered through Scienceware Flowmi 40-µm cell strainers (VWR). A haemocytometer and trypan blue staining were used to evaluate cell concentration and viability.

### scRNA-seq

According to the protocol of Chromium Single-cell 3′ Reagent V3 Kits manufacturer (10 × Genomics), the scRNA-seq libraries were prepared. Then, the single-cell suspensions were added to Chromium Single Cell Controller Instrument (10 × Genomics) to generate single-cell gel beads in emulsions (GEMs). In brief, 10^5^–10^6^ single cells suspended in 1 × PBS containing 0.04% BSA free with calcium and magnesium were added to each channel to generate GEMs, and reverse-transcription reactions were performed to generate barcoded full-length cDNA. After disruption of emulsions using the recovery agent, cDNA was purified with DynaBeads Myone Silane Beads (Thermo Fisher Scientific) and amplified by PCR. The amplification cycle depended on the recovered cells. The amplified cDNA was then fragmented, end-repaired, A-tailed, index and adaptor ligated, and library amplified. Finally, these libraries were sequenced on the Illumina sequencing platform (HiSeq X Ten), and 150 bp paired-end reads were obtained. In the above processes, each mRNA is randomly connected with a UMI (unique molecular identifier) after reverse transcription, and the number of mRNA can be counted through UMI. For the same mRNA, it is almost impossible to connect the same UMI, which can avoid the bias caused by PCR. All sequencing data were submitted to the BioProject of National Center for Biotechnology Information (NCBI) Sequence Read Archive (SRA) under accession numbers (SRR12791873 (AM_CTRL), SRR12791872 (AM_EM), SRR12791871 (AM_EC)).

### QC

The Cell Ranger Software Pipeline (Version 3.0.0) for demultiplexing of cellular barcodes is provided by 10 × Genomics. The STAR aligner (version 3.1.0) was used to map reads to the genome and transcriptome. Downsampling of mapped reads was required to generate normalized aggregate data across samples, producing a matrix of gene counts versus cells. We used the R package Seurat (version 3.1.1) [49] to process the UMI count matrix. Eliminating low-quality cells and likely multiplet captures are the key issues in microdroplet-based experiments. Theoretically, the number of genes and the number of UMIs will be concentrated in a certain region in most cells. According to their distribution characteristics, the distribution model can be fitted. Assuming a Gaussian distribution of the UMI/gene number per cell, we applied a criterion to filter the limit of the UMI/gene number beyond the mean ± two fold standard deviations (Additional file 1: Figure S1B). After visual inspection of the distribution of cells by the fraction of mitochondrial genes expressed, we further discarded low-quality cells in which > 25% of the counts belonged to mitochondrial genes. In total, 36,781 single cells were left by these QC criteria and were included in the following analysis. Library size normalization was performed in Seurat on the filtered matrix, and the normalized count was obtained.

For cells captured in sequencing, in order to avoid the bias brought by doublets, we set the threshold to 10% and detected the doublet cells according to the method recommended by Scrublet software [50]. Briefly, given a raw (unnormalized) UMI counts matrix counts_matrix with cells as rows and genes as columns, a k-nearest-neighbor classifier is used to calculate a continuous doublet_score (between 0 and 1) for each transcriptome. The score is automatically thresholded to generate predicted_doublets, a boolean array that is True for predicted doublets and False otherwise. The doublet score and predicted doublets were mapped to the *t*SNE map.

### Dimensional reduction, clustering and cell type identification

Identification of the most variable genes in a single cell was performed using the method described by Macosko [51]. In summary, the average expression and dispersion of each gene were first calculated, and then the genes were assigned to eight bins based on their expression. Principal component analysis (PCA) was performed to reduce the dimensionality of the log-transformed gene-barcode matrices of the top variable genes, and the cell clustering was based on the graph clustering method. In addition, two-dimensional visualization was performed by *t*-SNE method. To identify genes that were significantly differentially expressed between clusters, the likelihood ratio test was used to detect changes in both average expression and percentage of expressed cells. Therefore, we adopted a new calculation method for scRNA-seq unbiased cell type identification, named R Package SingleR (version 0.2.2), to independently infer the cell source and identify the cell type of each single cell by referring to the ‘Immgen’ data set [52].

The findMarker function of Seurat package was used to identify the DEGs. A P value < 0.05 and |log_2_foldchange|> 0.58 were set as the differential expression thresholds. The hypergeometric distribution of R package was used for GO enrichment and KEGG pathway enrichment analyses of DEGs.

### CNV analysis

The CNV levels were evaluated through the normalization of scRNA-seq gene expression arrays with the inferCNV R package (version 1.3.1) [53]. Genes were sorted according to their locations on chromosomes, and a window size containing 101 genes was used to determine the moving average of gene expression. The expression was then centred to zero by subtracting the mean. The T/NK cells, macrophages and master cells were selected as normal cells, while the remaining cells were selected as malignant cells. The final CNV profiles were obtained by denoising.

### Pseudotime analysis

Monocle2 package (version 2.8.0) was used to assay the evolutionary pseudotime [54]. The importCDS function in Monocle was used to convert the original count in the Seurat object into CellDataSet, and the differentialGeneTest function of Monocle2 package was used to select genes that may help cells sequence across the pseudotime trajectory (qval < 0.01). The dimension reduction function was used for clustering analysis, and then the orderCells function was used to infer the trajectory with default parameters. The pseudotime function (plot_genes_in_pseudotime) was used to map gene expression.

### HE staining

Samples from patients with AM or leiomyoma were fixed in 4% paraformaldehyde (PFA, Servicebio) for 48 h, embedded in paraffin, and sliced into 4-µm sections using a microtome. After deparaffinization and rehydration, the sections were stained with haematoxylin (Servicebio) for 5 min and then rinsed with distilled water. Next, eosin staining (Servicebio) was performed for 5 min, followed by gradient alcohol dehydration and submersion in xylene. Finally, neutral gum was used to seal the slides, and pathological changes were observed under light microscopy (Nikon Eclipse E100, Japan).

### Immunofluorescence staining analysis

Sample sections were deparaffinized in xylene and dehydrated in ethanol with a decreasing concentration gradient, following antigen retrieval in EDTA antigen retrieval solution (PH 8.0, Servicebio) by boiling the sections for 20 min. Endogenous peroxidase activity was blocked with 3% H_2_O_2_ for 25 min. To avoid nonspecific binding, slices were blocked with 3% BSA (Servicebio) for 30 min at RT. Then, the primary antibodies rabbit pAb anti-PECAM1 (dilution 1:100, Servicebio) and mouse mAb anti-EPCAM (dilution 1:200, Servicebio) were used in immunofluorescence staining analysis, and the sections were incubated with appropriate primary antibodies at 4 °C overnight, followed by washing with PBS 3 times. Then, the slices were incubated with Cy3-conjugated goat anti-rabbit IgG (dilution 1:200, Servicebio) or Alexa Fluor 488-conjugated goat anti-mouse IgG (dilution 1:200, Servicebio) at RT for 50 min. The nuclei were stained with DAPI (Servicebio) for 10 min and then washed with PBS 3 times. Finally, sections were imaged with a fluorescence microscope (Nikon Eclipse C1, Japan).

### CD34-PAS double staining

Paraffin sections were routinely deparaffinized and dehydrated in xylene and ethanol solutions. Antigen retrieval was performed in citrate antigen retrieval buffer (PH 6.0, Servicebio) at 100 °C for 20 min. Subsequently, sections were placed in 3% hydrogen peroxide solution to block endogenous peroxidase for 25 min at RT and washed with PBS. BSA (3%) was added to the sections for 30 min at RT to block nonspecific binding sites. Then, the sections were incubated with anti-CD34 (dilution 1:500, Servicebio) at 4 °C overnight. After washing three times with PBS, sections were incubated with HRP-labelled goat anti-rabbit IgG for 50 min at RT. Next, 3,3′-diaminobenzidine tetrahydrochloride (DAB, Servicebio) was used to stain the positive brown-yellow reactions. After the sections were washed with running water, they were placed in periodic acid solution for 20 min in a dark environment. Finally, the nuclei were countersigned with haematoxylin for 3 min, rinsed with running water, and mounted with neutral gum. VM channels were positively stained with PAS but not CD34.

### Statistical analysis

The results were evaluated using GraphPad Prism 5.0 software (GraphPad Software, Inc.), and the data were analysed by one‑way ANOVA followed by a post-hoc Tukey test and presented as the mean ± SD. Differences were considered to be significant when the P value < 0.05.

## Supplementary Information

**Additional file 1: Figure S1. **HE staining and QC. (A) Samples from the AM_CTRL, AM_EM and AM_EC groups were HE stained, and the black arrow shows the gland invading the muscular layer. (B) X axis represents the number of UMI in each cell, and y-axis represents the number of genes in each cell. The distribution model is fitted according to the linear relationship. Yellow dots indicate cells that deviate from the threshold and will be removed in subsequent analysis. (C) The doublet score increases gradually from light color to dark blue (left figure), the red dots represent doubles predicted by Scrublet (right figure). The proportion of mitochondrial genes (D), the number of genes expressed (E), and the number of UMIs (F) in each cell before and after QC are shown in the violin plots. (G) The mean proportion of mitochondrial genes, mean number of genes expressed, mean number of UMIs in each cell and cell number of the three sample groups before and after QC are shown. HE, Hematoxylin-Eosin; QC, Quality Control. **Figure S2. **Cell type identification and heatmap of gene expression in clusters. (A) Seventeen clusters were displayed in the AM_CTRL, EAM_EM and AM_EC groups. (B) Heatmap showing the expression levels of specific markers in each cluster. (C) The cell number and percentage corresponding to each cell type were counted. Complementary representative markers of different cell types (D) and corresponding violin plots (E) are shown. **Figure S3 **Colocalization of epithelial cell and endothelial cell markers in cluster 1. (A, B) Complementary epithelial cell markers (CDH1 and KRT7), endothelial cell markers (VWF and CDH5) and colocalization of the two cell type markers are displayed in the *t*-SNE map. (C) Confirmatory colocalization of EPCAM and PECAM1 was conducted in additional AM_CTRL (V) (n=3), AM_EM (V) (n=3), AM_EC (V) (n=3) samples, EPCAM (red), PECAM1 (green) and nuclei (blue) were stained. The white arrows show the colocalized cells containing EPCAM and PECAM1, scale bars = 20 μm, *P < 0.05. GO (D) and KEGG (E) analyses of upregulated genes in cluster 1 compared with all other clusters (cluster 2 to cluster 17). Representative GO (F) and KEGG (G) terms of upregulated genes in cluster 1 compared with the epithelial cell population (cluster 7 and cluster 17) are displayed. GO, Gene Ontology; KEGG, Kyoto Encyclopedia of Genes and Genomes. **Figure S4 **Gene expression changes and functional analyses of the AM_CTRL, AM_EM and AM_EC groups. Representative GO (A) and KEGG (C) terms of upregulated DEGs in the AM_EC group compared with the AM_EM group are shown. Representative GO (B) and KEGG (D) terms of upregulated genes in the AM_EM group compared with those in the AM_CTRL group are shown. (E) Venn diagram showing the common change genes between AM_EM versus AM_CTRL and AM_EC versus AM_EM. (F) Enriched GO terms of common changed genes in (E) are shown. GO, Gene Ontology; KEGG, Kyoto Encyclopedia of Genes and Genomes. **Figure S5.** Identification of subclusters in epithelial cell populations. (A) The *t*-SNE map shows 7 subclusters in the epithelial cell group. Histograms show the proportion of subcluster 1 in AM_EM and AM_EC (B), and the proportion of subcluster 4 is shown in (C). (D) The heatmap displays the expression levels of specific markers in each subcluster. Representative GO (E) and KEGG (F) terms of the top 500 genes expressed in subcluster 1 are indicated. The corresponding GO (G) and KEGG (H) analyses of subcluster 4 are also presented. GO, Gene Ontology; KEGG, Kyoto Encyclopedia of Genes and Genomes. **Figure S6.** Change in marker distribution on the pseudotime trajectory and verification of VM formation. (A) Distribution of groups on the pseudotime trajectory. (B) Cell type, including epithelial cell, cluster 1 and endothelial cell, distributed on the pseudotime trajectory. (C, D) Epithelial cell markers (EPCAM, CDH1 and KRT7) and endothelial cell markers (PECAM1, VWF and CDH5) distributed on the pseudotime trajectory. (E) Confirmatory VM channel formations were conducted in additional AM_CTRL (V) (n=3), AM_EM (V) (n=3), AM_EC (V) (n=3) samples, VM channels were positive for PAS staining but negative for CD34 (black arrows); red arrows represent channels positive for CD34; scale bars = 50 μm. (F) Histogram indicates the number of VM channels in different groups; ns represents not significant, *P < 0.05. VM, Vasculogenic Mimicry. PAS, Periodic Acid-Schiff.

**Additional file 2: Table S1.** Doublet score and predicted doublets.

**Additional file 3: Table S2.** DEGs of AM_EC vs. AM_EM (all cell types).

**Additional file 4: Table S3.** Coexisting genes in Venn.

**Additional file 5: Table S4.** DEGs of AM_EC vs. AM_EM (epithelial cells).

**Additional file 6: Table S5.** Clinical characteristics of AM patients and hysteromyoma samples used in this study.

## Data Availability

The datasets used and/or analyzed during the current study are available in the additional files. All sequencing data were submitted to the BioProject of National Center for Biotechnology Information (NCBI) Sequence Read Archive (SRA) under accession numbers (SRR12791873 (AM_CTRL), SRR12791872 (AM_EM), SRR12791871 (AM_EC)).

## Abbreviations

AM: Adenomyosis
CMI terms: Cancer-, cell motility- and inflammation-associated terms
CNV: Copy number variation
DAB: 3,3′-Diaminobenzidine tetrahydrochloride
DEGs: Differentially expressed genes
EET: Epithelial-endothelial transition
EMT: Epithelial-mesenchymal transition
GEMs: Gel beads in emulsions
GnRHa: Gonadotrophin-releasing hormone agonist
GO: Gene ontology
HE: Hematoxylin–eosin
KEGG: Kyoto encyclopedia of genes and genomes
MRI: Magnetic resonance imaging
PAS: Periodic acid-Schiff
QC: Quality control
RCF: Relative centrifugal force
RT: Room temperature
scRNA-seq: Single-cell RNA sequencing
*t*-SNE: *t*-distributed stochastic neighbor embedding
TVUS: Transvaginal ultrasound scan
UMI: Unique molecular identifier
VM: Vasculogenic mimicry
