# Supplementary figures and images for: Single-cell transcriptomic analysis of eutopic endometrium and ectopic lesions of adenomyosis
Source: Cell Biosci. 2021 Mar 8;11:51. doi: 10.1186/s13578-021-00562-z (PMC7938473; doi:10.1186/s13578-021-00562-z)

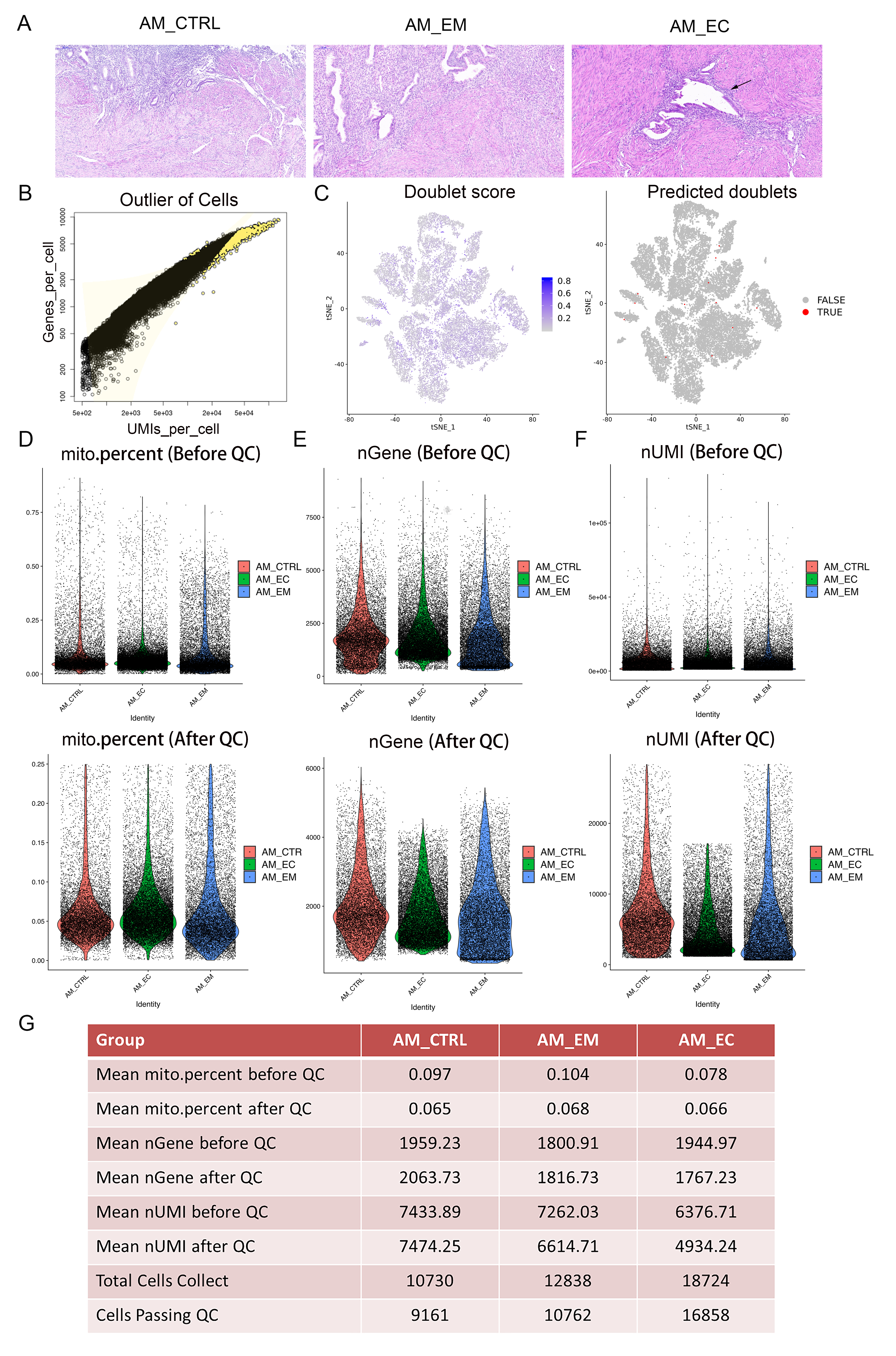

Supplement: Supplementary file 1 — Additional file 1: Figure S1. HE staining and QC. (A) Samples from the AM_CTRL, AM_EM and AM_EC groups were HE stained, and the black arrow shows the gland invading the muscular layer. (B) X axis represents the number of UMI in each cell, and y-axis represents the number of genes in each cell. The distribution model is fitted according to the linear relationship. Yellow dots indicate cells that deviate from the threshold and will be removed in subsequent analysis. (C) The doublet score increases gradually from light color to dark blue (left figure), the red dots represent doubles predicted by Scrublet (right figure). The proportion of mitochondrial genes (D), the number of genes expressed (E), and the number of UMIs (F) in each cell before and after QC are shown in the violin plots. (G) The mean proportion of mitochondrial genes, mean number of genes expressed, mean number of UMIs in each cell and cell number of the three sample groups before and after QC are shown. HE, Hematoxylin-Eosin; QC, Quality Control. Figure S2. Cell type identification and heatmap of gene expression in clusters. (A) Seventeen clusters were displayed in the AM_CTRL, EAM_EM and AM_EC groups. (B) Heatmap showing the expression levels of specific markers in each cluster. (C) The cell number and percentage corresponding to each cell type were counted. Complementary representative markers of different cell types (D) and corresponding violin plots (E) are shown. Figure S3 Colocalization of epithelial cell and endothelial cell markers in cluster 1. (A, B) Complementary epithelial cell markers (CDH1 and KRT7), endothelial cell markers (VWF and CDH5) and colocalization of the two cell type markers are displayed in the t-SNE map. (C) Confirmatory colocalization of EPCAM and PECAM1 was conducted in additional AM_CTRL (V) (n=3), AM_EM (V) (n=3), AM_EC (V) (n=3) samples, EPCAM (red), PECAM1 (green) and nuclei (blue) were stained. The white arrows show the colocalized cells containing EPCAM and PECAM1, sc [file 13578_2021_562_MOESM1_ESM.zip › Figure S1.jpg]

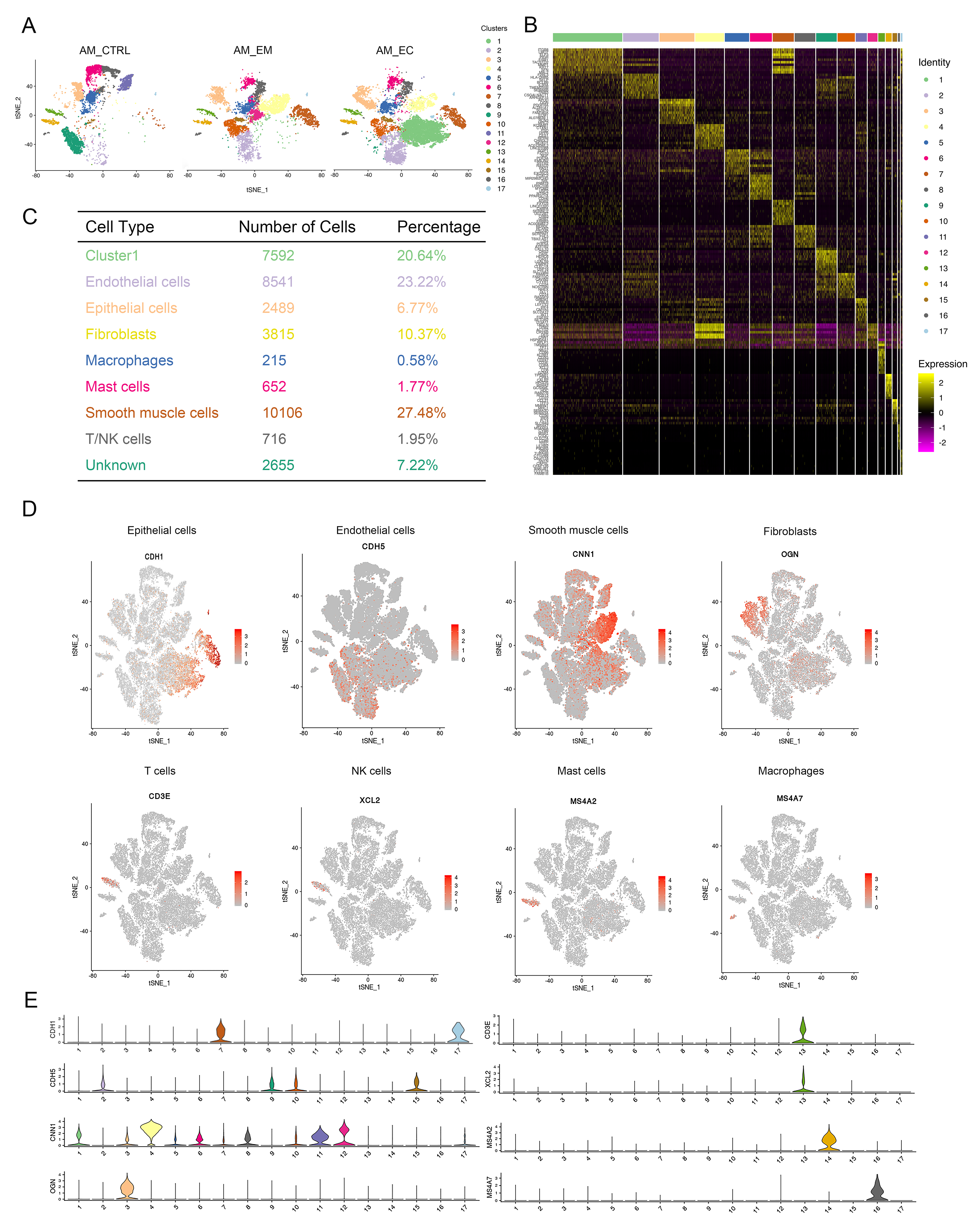

Supplement: Supplementary file 1 — Additional file 1: Figure S1. HE staining and QC. (A) Samples from the AM_CTRL, AM_EM and AM_EC groups were HE stained, and the black arrow shows the gland invading the muscular layer. (B) X axis represents the number of UMI in each cell, and y-axis represents the number of genes in each cell. The distribution model is fitted according to the linear relationship. Yellow dots indicate cells that deviate from the threshold and will be removed in subsequent analysis. (C) The doublet score increases gradually from light color to dark blue (left figure), the red dots represent doubles predicted by Scrublet (right figure). The proportion of mitochondrial genes (D), the number of genes expressed (E), and the number of UMIs (F) in each cell before and after QC are shown in the violin plots. (G) The mean proportion of mitochondrial genes, mean number of genes expressed, mean number of UMIs in each cell and cell number of the three sample groups before and after QC are shown. HE, Hematoxylin-Eosin; QC, Quality Control. Figure S2. Cell type identification and heatmap of gene expression in clusters. (A) Seventeen clusters were displayed in the AM_CTRL, EAM_EM and AM_EC groups. (B) Heatmap showing the expression levels of specific markers in each cluster. (C) The cell number and percentage corresponding to each cell type were counted. Complementary representative markers of different cell types (D) and corresponding violin plots (E) are shown. Figure S3 Colocalization of epithelial cell and endothelial cell markers in cluster 1. (A, B) Complementary epithelial cell markers (CDH1 and KRT7), endothelial cell markers (VWF and CDH5) and colocalization of the two cell type markers are displayed in the t-SNE map. (C) Confirmatory colocalization of EPCAM and PECAM1 was conducted in additional AM_CTRL (V) (n=3), AM_EM (V) (n=3), AM_EC (V) (n=3) samples, EPCAM (red), PECAM1 (green) and nuclei (blue) were stained. The white arrows show the colocalized cells containing EPCAM and PECAM1, sc [file 13578_2021_562_MOESM1_ESM.zip › Supplementary Figure S2.jpg]

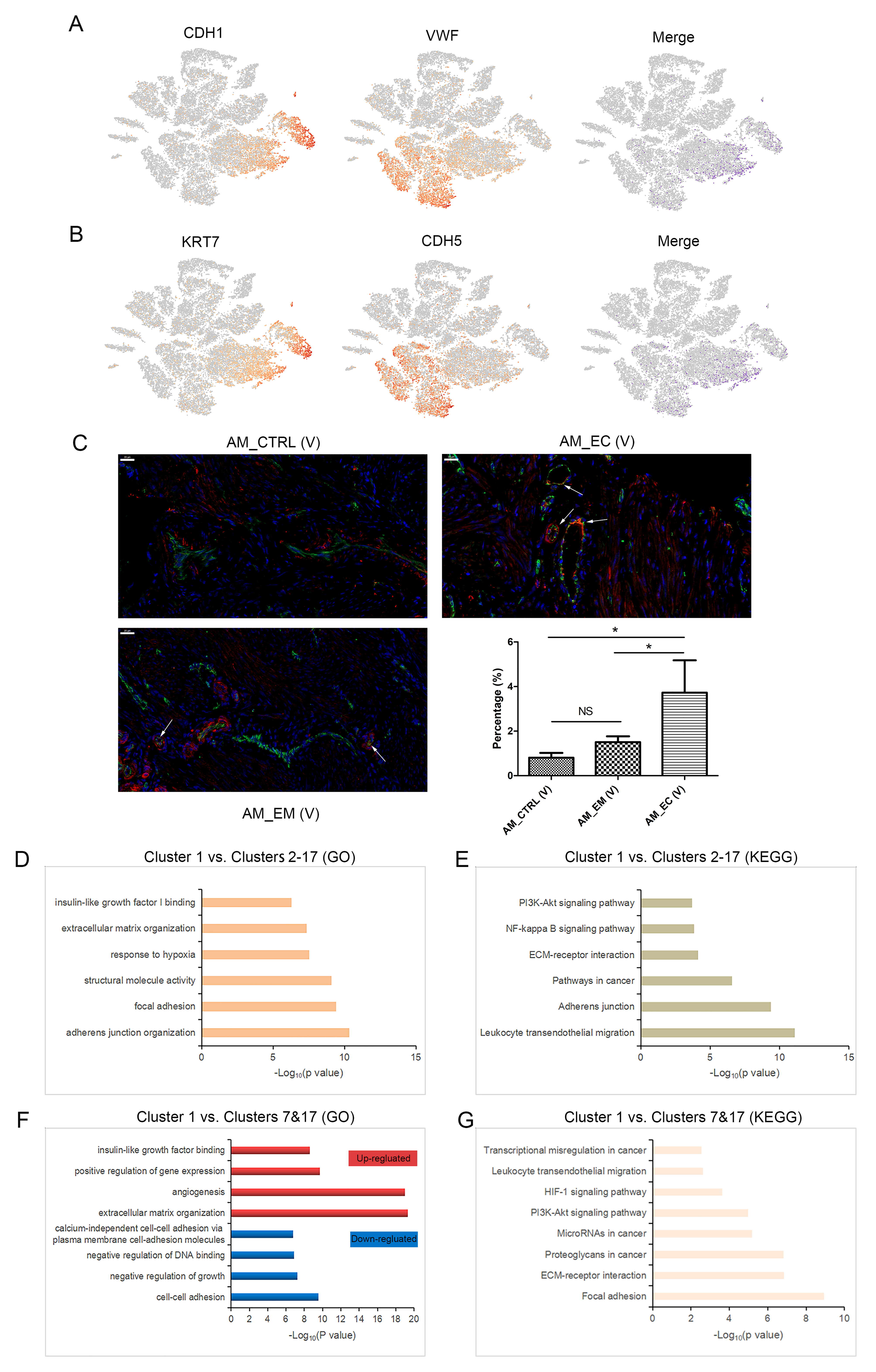

Supplement: Supplementary file 1 — Additional file 1: Figure S1. HE staining and QC. (A) Samples from the AM_CTRL, AM_EM and AM_EC groups were HE stained, and the black arrow shows the gland invading the muscular layer. (B) X axis represents the number of UMI in each cell, and y-axis represents the number of genes in each cell. The distribution model is fitted according to the linear relationship. Yellow dots indicate cells that deviate from the threshold and will be removed in subsequent analysis. (C) The doublet score increases gradually from light color to dark blue (left figure), the red dots represent doubles predicted by Scrublet (right figure). The proportion of mitochondrial genes (D), the number of genes expressed (E), and the number of UMIs (F) in each cell before and after QC are shown in the violin plots. (G) The mean proportion of mitochondrial genes, mean number of genes expressed, mean number of UMIs in each cell and cell number of the three sample groups before and after QC are shown. HE, Hematoxylin-Eosin; QC, Quality Control. Figure S2. Cell type identification and heatmap of gene expression in clusters. (A) Seventeen clusters were displayed in the AM_CTRL, EAM_EM and AM_EC groups. (B) Heatmap showing the expression levels of specific markers in each cluster. (C) The cell number and percentage corresponding to each cell type were counted. Complementary representative markers of different cell types (D) and corresponding violin plots (E) are shown. Figure S3 Colocalization of epithelial cell and endothelial cell markers in cluster 1. (A, B) Complementary epithelial cell markers (CDH1 and KRT7), endothelial cell markers (VWF and CDH5) and colocalization of the two cell type markers are displayed in the t-SNE map. (C) Confirmatory colocalization of EPCAM and PECAM1 was conducted in additional AM_CTRL (V) (n=3), AM_EM (V) (n=3), AM_EC (V) (n=3) samples, EPCAM (red), PECAM1 (green) and nuclei (blue) were stained. The white arrows show the colocalized cells containing EPCAM and PECAM1, sc [file 13578_2021_562_MOESM1_ESM.zip › Supplementary Figure S3.jpg]

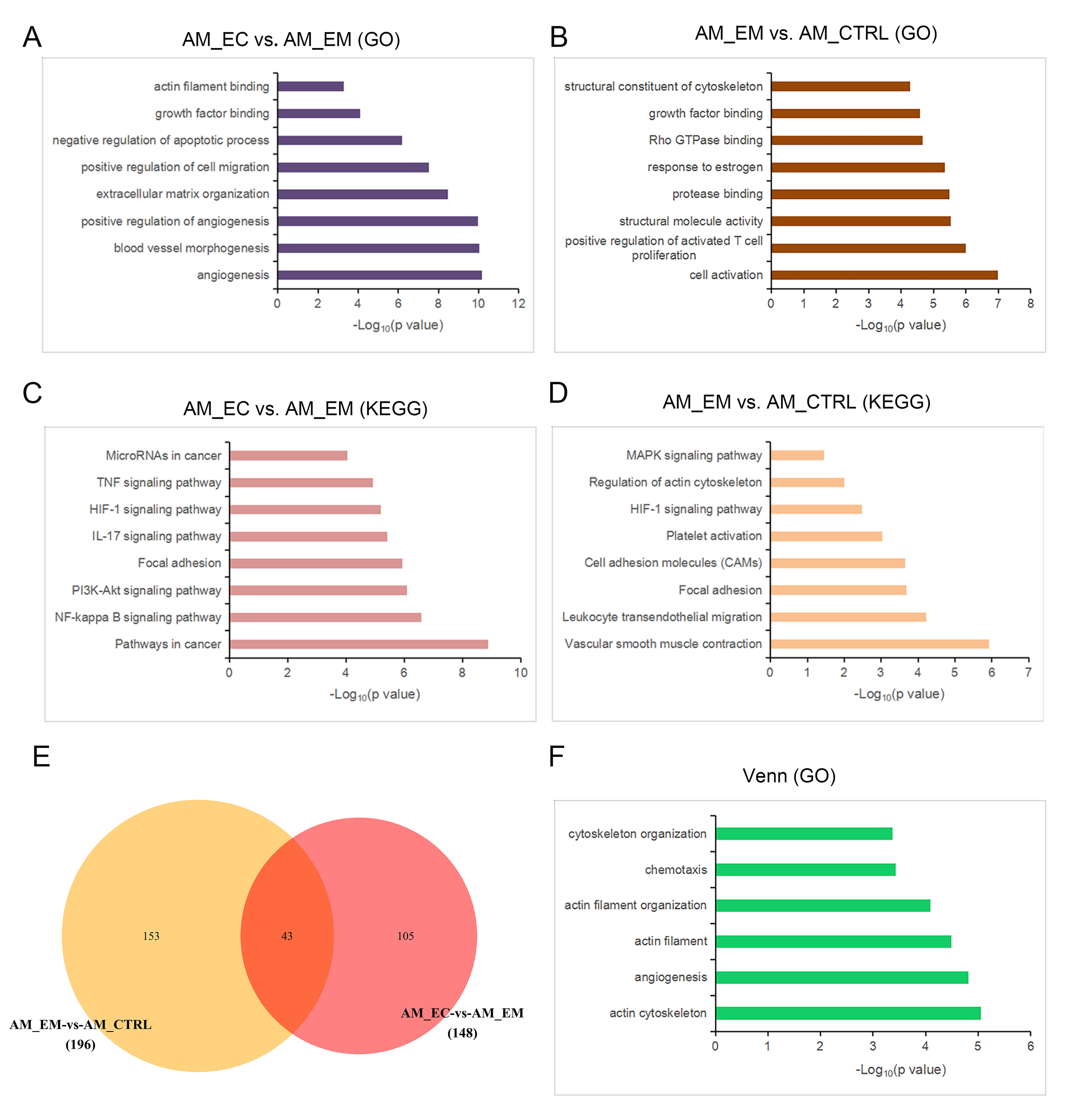

Supplement: Supplementary file 1 — Additional file 1: Figure S1. HE staining and QC. (A) Samples from the AM_CTRL, AM_EM and AM_EC groups were HE stained, and the black arrow shows the gland invading the muscular layer. (B) X axis represents the number of UMI in each cell, and y-axis represents the number of genes in each cell. The distribution model is fitted according to the linear relationship. Yellow dots indicate cells that deviate from the threshold and will be removed in subsequent analysis. (C) The doublet score increases gradually from light color to dark blue (left figure), the red dots represent doubles predicted by Scrublet (right figure). The proportion of mitochondrial genes (D), the number of genes expressed (E), and the number of UMIs (F) in each cell before and after QC are shown in the violin plots. (G) The mean proportion of mitochondrial genes, mean number of genes expressed, mean number of UMIs in each cell and cell number of the three sample groups before and after QC are shown. HE, Hematoxylin-Eosin; QC, Quality Control. Figure S2. Cell type identification and heatmap of gene expression in clusters. (A) Seventeen clusters were displayed in the AM_CTRL, EAM_EM and AM_EC groups. (B) Heatmap showing the expression levels of specific markers in each cluster. (C) The cell number and percentage corresponding to each cell type were counted. Complementary representative markers of different cell types (D) and corresponding violin plots (E) are shown. Figure S3 Colocalization of epithelial cell and endothelial cell markers in cluster 1. (A, B) Complementary epithelial cell markers (CDH1 and KRT7), endothelial cell markers (VWF and CDH5) and colocalization of the two cell type markers are displayed in the t-SNE map. (C) Confirmatory colocalization of EPCAM and PECAM1 was conducted in additional AM_CTRL (V) (n=3), AM_EM (V) (n=3), AM_EC (V) (n=3) samples, EPCAM (red), PECAM1 (green) and nuclei (blue) were stained. The white arrows show the colocalized cells containing EPCAM and PECAM1, sc [file 13578_2021_562_MOESM1_ESM.zip › Supplementary Figure S4.jpg]

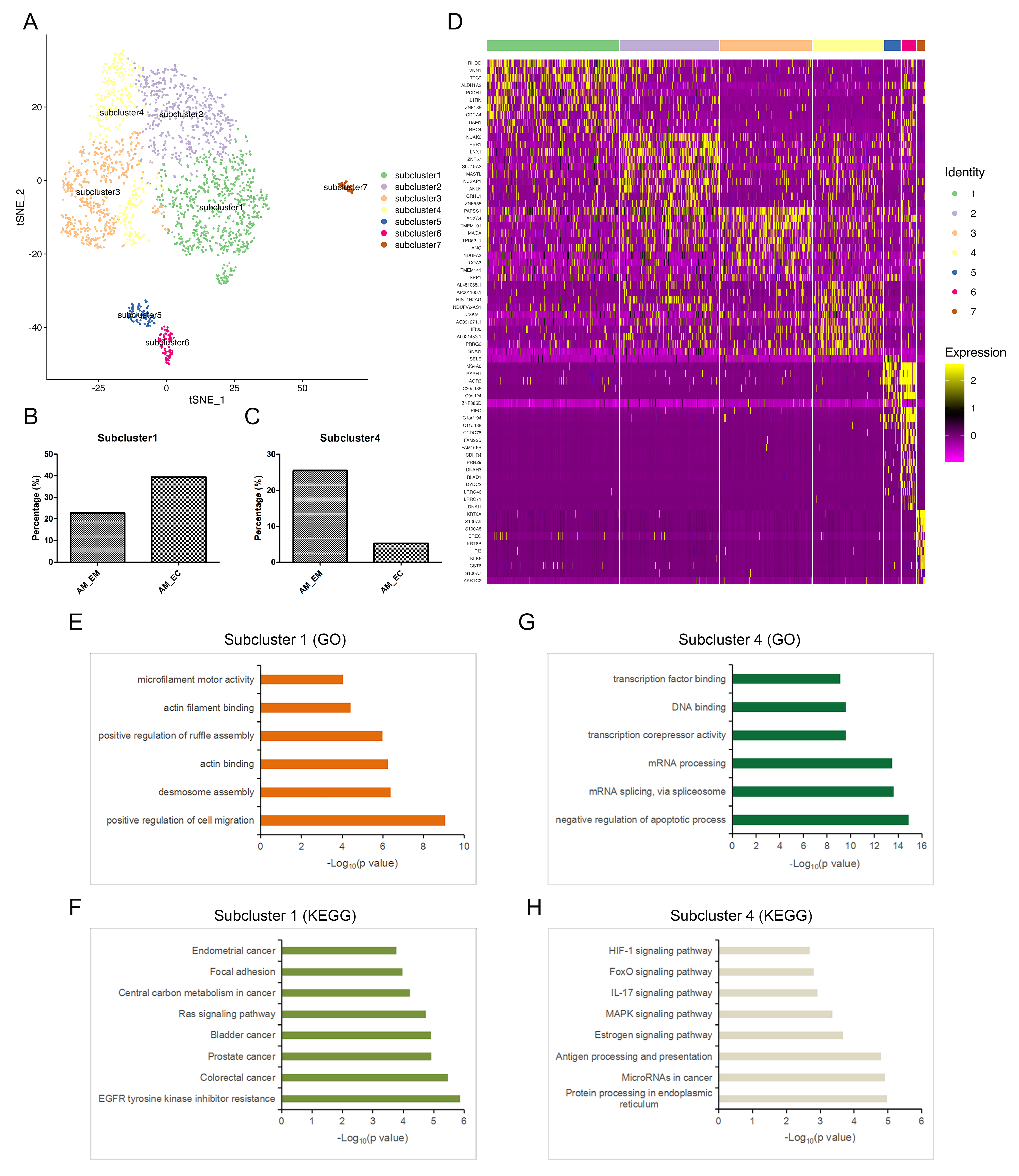

Supplement: Supplementary file 1 — Additional file 1: Figure S1. HE staining and QC. (A) Samples from the AM_CTRL, AM_EM and AM_EC groups were HE stained, and the black arrow shows the gland invading the muscular layer. (B) X axis represents the number of UMI in each cell, and y-axis represents the number of genes in each cell. The distribution model is fitted according to the linear relationship. Yellow dots indicate cells that deviate from the threshold and will be removed in subsequent analysis. (C) The doublet score increases gradually from light color to dark blue (left figure), the red dots represent doubles predicted by Scrublet (right figure). The proportion of mitochondrial genes (D), the number of genes expressed (E), and the number of UMIs (F) in each cell before and after QC are shown in the violin plots. (G) The mean proportion of mitochondrial genes, mean number of genes expressed, mean number of UMIs in each cell and cell number of the three sample groups before and after QC are shown. HE, Hematoxylin-Eosin; QC, Quality Control. Figure S2. Cell type identification and heatmap of gene expression in clusters. (A) Seventeen clusters were displayed in the AM_CTRL, EAM_EM and AM_EC groups. (B) Heatmap showing the expression levels of specific markers in each cluster. (C) The cell number and percentage corresponding to each cell type were counted. Complementary representative markers of different cell types (D) and corresponding violin plots (E) are shown. Figure S3 Colocalization of epithelial cell and endothelial cell markers in cluster 1. (A, B) Complementary epithelial cell markers (CDH1 and KRT7), endothelial cell markers (VWF and CDH5) and colocalization of the two cell type markers are displayed in the t-SNE map. (C) Confirmatory colocalization of EPCAM and PECAM1 was conducted in additional AM_CTRL (V) (n=3), AM_EM (V) (n=3), AM_EC (V) (n=3) samples, EPCAM (red), PECAM1 (green) and nuclei (blue) were stained. The white arrows show the colocalized cells containing EPCAM and PECAM1, sc [file 13578_2021_562_MOESM1_ESM.zip › Supplementary Figure S5.jpg]

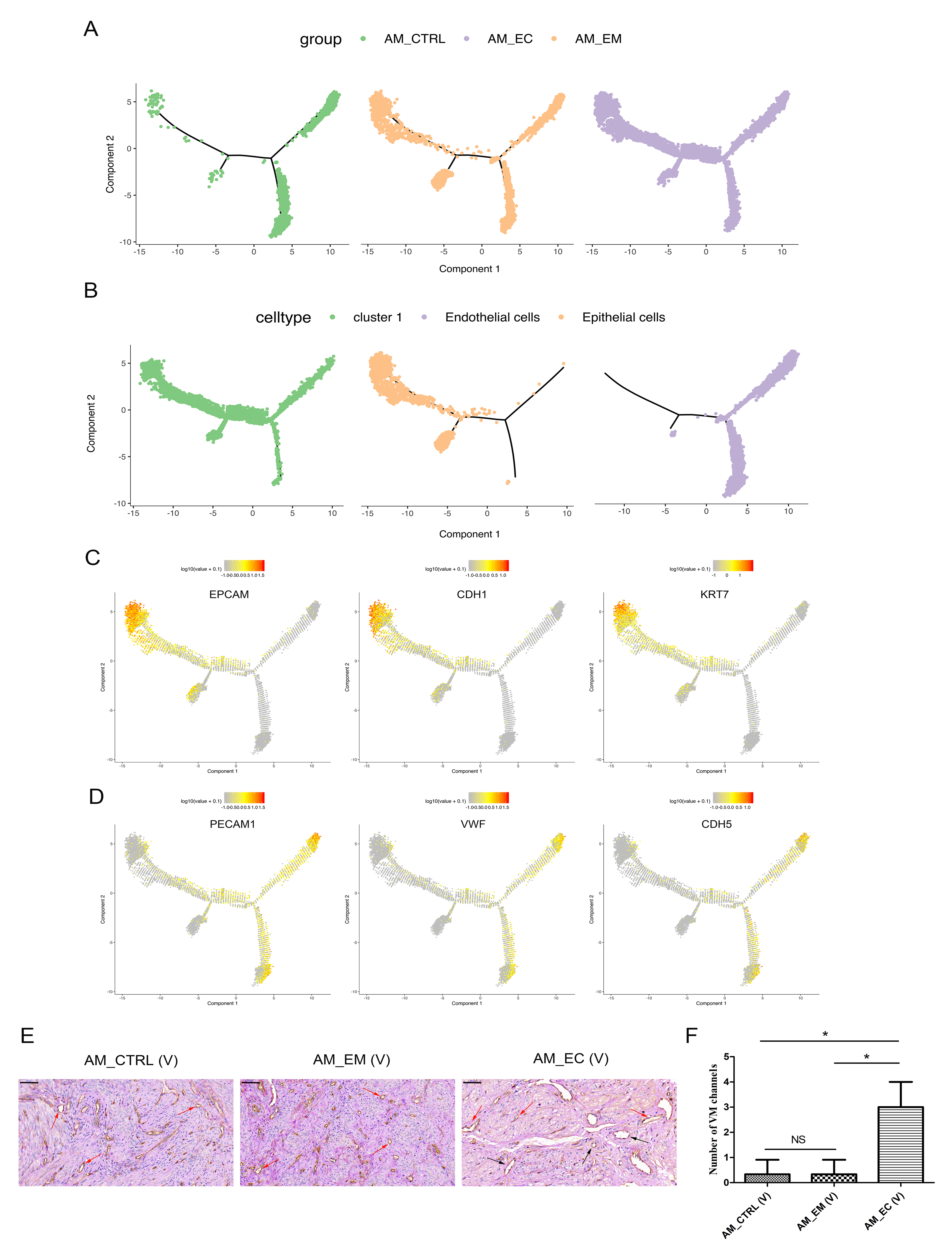

Supplement: Supplementary file 1 — Additional file 1: Figure S1. HE staining and QC. (A) Samples from the AM_CTRL, AM_EM and AM_EC groups were HE stained, and the black arrow shows the gland invading the muscular layer. (B) X axis represents the number of UMI in each cell, and y-axis represents the number of genes in each cell. The distribution model is fitted according to the linear relationship. Yellow dots indicate cells that deviate from the threshold and will be removed in subsequent analysis. (C) The doublet score increases gradually from light color to dark blue (left figure), the red dots represent doubles predicted by Scrublet (right figure). The proportion of mitochondrial genes (D), the number of genes expressed (E), and the number of UMIs (F) in each cell before and after QC are shown in the violin plots. (G) The mean proportion of mitochondrial genes, mean number of genes expressed, mean number of UMIs in each cell and cell number of the three sample groups before and after QC are shown. HE, Hematoxylin-Eosin; QC, Quality Control. Figure S2. Cell type identification and heatmap of gene expression in clusters. (A) Seventeen clusters were displayed in the AM_CTRL, EAM_EM and AM_EC groups. (B) Heatmap showing the expression levels of specific markers in each cluster. (C) The cell number and percentage corresponding to each cell type were counted. Complementary representative markers of different cell types (D) and corresponding violin plots (E) are shown. Figure S3 Colocalization of epithelial cell and endothelial cell markers in cluster 1. (A, B) Complementary epithelial cell markers (CDH1 and KRT7), endothelial cell markers (VWF and CDH5) and colocalization of the two cell type markers are displayed in the t-SNE map. (C) Confirmatory colocalization of EPCAM and PECAM1 was conducted in additional AM_CTRL (V) (n=3), AM_EM (V) (n=3), AM_EC (V) (n=3) samples, EPCAM (red), PECAM1 (green) and nuclei (blue) were stained. The white arrows show the colocalized cells containing EPCAM and PECAM1, sc [file 13578_2021_562_MOESM1_ESM.zip › Supplementary Figure S6.jpg]
